# TGR5 agonists induce peripheral and central hypersensitivity to bladder distension

**DOI:** 10.1038/s41598-022-14195-w

**Published:** 2022-06-15

**Authors:** Ashlee Caldwell, Luke Grundy, Andrea M. Harrington, Sonia Garcia-Caraballo, Joel Castro, Nigel W. Bunnett, Stuart M. Brierley

**Affiliations:** 1 Visceral Pain Research Group, College of Medicine and Public Health, Flinders Health and Medical Research Institute (FHMRI), Flinders University, Bedford Park, South Australia 5042 Australia; 2 Hopwood Centre for Neurobiology, Lifelong Health Theme, Level 7, South Australian Health and Medical Research Institute (SAHMRI), North Terrace, Adelaide, South Australia 5000 Australia; 3 Discipline of Medicine, University of Adelaide, Level 7, SAHMRI, North Terrace, Adelaide, South Australia 5000 Australia; 4 Department of Molecular Pathobiology, Department of Neuroscience and Physiology, Neuroscience Institute, New York University, New York, NY USA

**Keywords:** Ion channels in the nervous system, Peripheral nervous system, Bladder, Urogenital diseases, Neurophysiology

## Abstract

The mechanisms underlying chronic bladder conditions such as interstitial cystitis/bladder pain syndrome (IC/BPS) and overactive bladder syndrome (OAB) are incompletely understood. However, targeting specific receptors mediating neuronal sensitivity to specific stimuli is an emerging treatment strategy. Recently, irritant-sensing receptors including the bile acid receptor TGR5, have been identified within the viscera and are thought to play a key role in neuronal hypersensitivity. Here, in mice, we identify mRNA expression of TGR5 (*Gpbar1*) in all layers of the bladder as well as in the lumbosacral dorsal root ganglia (DRG) and in isolated bladder-innervating DRG neurons. In bladder-innervating DRG neurons *Gpbar1* mRNA was 100% co-expressed with *Trpv1* and 30% co-expressed with *Trpa1*. In vitro live-cell calcium imaging of bladder-innervating DRG neurons showed direct activation of a sub-population of bladder-innervating DRG neurons with the synthetic TGR5 agonist CCDC, which was diminished in *Trpv1*^*−/−*^ but not *Trpa1*^−/−^ DRG neurons. CCDC also activated a small percentage of non-neuronal cells. Using an ex vivo mouse bladder afferent recording preparation we show intravesical application of endogenous (5α-pregnan-3β-ol-20-one sulphate, Pg5α) and synthetic (CCDC) TGR5 agonists enhanced afferent mechanosensitivity to bladder distension. Correspondingly, in vivo intravesical administration of CCDC increased the number of spinal dorsal horn neurons that were activated by bladder distension. The enhanced mechanosensitivity induced by CCDC ex vivo and in vivo was absent using *Gpbar1*^−/−^ mice. Together, these results indicate a role for the TGR5 receptor in mediating bladder afferent hypersensitivity to distension and thus may be important to the symptoms associated with IC/BPS and OAB.

## Introduction

Chronic bladder pathologies such as interstitial cystitis/bladder pain syndrome (IC/BPS) and overactive bladder syndrome (OAB) affect up to 16% of the population, significantly decreasing the quality of life for sufferers^1–4^. Although there are important differences between these conditions, the common symptoms of frequency, urgency and discomfort^1,5^ are strongly indicative of neuronal hypersensitivity within bladder sensory pathways. Treatment options for each of these conditions are limited, suggesting a more complete understanding of the mechanisms underlying bladder sensory signalling is required.

The primary sensory afferents innervating the urinary bladder have cell bodies within the dorsal root ganglia (DRG)^6,7^. Sensory nerve endings for these afferent neurons are located throughout all layers of the bladder wall including the detrusor smooth muscle, the mucosal/interstitial tissue, and the urothelium^8,9^. Activation of these sensory nerve endings by various mechanical, chemical, or inflammatory stimuli results in sensory input into the dorsal horn of the spinal cord, where the bladder afferent neurons terminate^6,10,11^. From here, signals are relayed into autonomic and central pathways via activation of second order neurons, facilitating bladder relaxation and conscious sensory perception, respectively^11,12^. The specificity of the sensory input into central pathways is driven by the combination of receptors and ion channels expressed on the sensory terminals, allowing for the differentiation of distinct stimuli. Specific targeting of receptors on sensory nerve endings therefore presents an opportunity to modulate the intensity of bladder sensory signals. Pharmacological modulation of receptors is an invaluable method of treatment development for a variety of conditions, in particular G-protein coupled receptors (GPCRs) constitute the majority of current and potential pharmaceutical receptor targets^13^. This includes targets to treat itch, which can be induced by both histaminergic and non-histaminergic mechanisms, the latter of which includes bile acids and agonists of Mas-gene-related G protein-coupled receptors (Mrgprs)^14^.

Itch-inducing GPCRs have recently been highlighted as potential pharmacological targets in visceral hypersensitivity conditions^15^. We have demonstrated that activation of histamine receptor H_1_R, or the Mrgpr subtypes MrgprA3 and MrgprC11 modulate bladder afferent sensitivity to distension^16,17^. Takeda G-Protein Receptor 5 (TGR5), also known as Membrane-Bile Acid Receptor (M-BAR) or G-protein-coupled Bile Acid Receptor 1 (GPBAR1), is a membrane-bound G-protein coupled bile acid receptor^18,19^. TGR5 is expressed throughout the viscera and the nervous system, including the gastrointestinal tract^20–22^, the bladder^23^, as well as neurons and glial cells in the DRG^24–26^. TGR5 has been recently proposed as the integral receptor required for pruritus associated with chronic and pregnancy-related liver diseases, with TGR5 activation in DRG neurons resulting in neuronal hyperexcitability and the release of neurotransmitters in the dorsal horn^20,24,26^. Furthermore, TGR5 has been implicated in the pathology of irritable bowel syndrome (IBS), a common co-morbidity of OAB and IC/BPS^26^. In addition to bile acids, TGR5 can be activated by neurosteroids such as progesterone and progesterone metabolites^27,28^ to evoke itch sensations via a TGR5-dependent mechanism^28,29^. Progesterone metabolites are conjugated and excreted via the urine, while bile acids have also been identified in the urine^30–34^. However, the role of TGR5 in regulating bladder afferent hypersensitivity is currently unknown.

Given the presence of endogenous TGR5 agonists in the bladder and the implicated roles for TGR5 in irritant sensation, we sought to investigate the expression and function of TGR5 in bladder afferent sensitivity. Here we show that TGR5 is expressed in a sub-population of bladder-innervating DRG neurons that co-express TRPV1, and to a lesser extent TRPA1. Endogenous and synthetic TGR5 agonists enhanced bladder afferent responses to bladder distension both ex vivo and in vivo, effects which are lost in mice lacking the TGR5 receptor (*Gpbar1*^−/−^). This study shows for the first time the ability of TGR5 to regulate bladder afferent mechanosensitivity.

## Methods

### Ex vivo bladder afferent nerve recordings

Nerve recordings were performed in an ex vivo model as previously described^16,17,35–37^. Mice were humanely killed via CO_2_ inhalation, and the entire lower abdomen was removed and submerged in a modified organ bath under continual perfusion with gassed (95% O_2_ and 5% CO_2_) Krebs-bicarbonate solution (composition in mmol/L: 118.4 NaCl, 24.9 NaHCO_3_, 1.9 CaCl_2_, 1.2 MgSO_4_, 4.7 KCl, 1.2 KH_2_PO_4_, 11.7 glucose) at 35 °C. The bladder, urethra, and ureters were exposed by removing excess tissue. Ureters were tied with 4–0 perma-hand silk (Ethicon, #LA53G). The bladder was catheterised (PE 50 tubing) via the urethra and connected to a syringe pump (NE-1000) to allow a controlled fill rate of 30 µL/min with saline (NaCl, 0.9%). To enable intravesical pressure recording during graded distension, a second catheter was inserted through the dome of the bladder, secured with silk, and connected to a pressure transducer (NL108T2; Digitimer). Pelvic nerves, isolated from all other nerve fibres between the pelvic ganglia and the spinal cord, were dissected into fine multiunit branches and a single branch was placed within a sealed glass pipette containing a microelectrode (WPI) attached to a Neurolog headstage (NL100AK; Digitimer). Nerve activity was amplified (NL104), filtered (NL 125/126, band pass 50–5000 Hz, Neurolog; Digitimer), and digitised (CED 1401; Cambridge Electronic Design, Cambridge, UK) to a PC for offline analysis using Spike2 software (Cambridge Electronic Design, Cambridge, UK). The number of action potentials crossing a pre-set threshold at twice the background electrical noise was determined per second to quantify the afferent response. Single-unit analysis was performed offline using Spike2 software by matching individual spike waveforms through linear interpolation. A single unit was deemed to be responsive if a greater than 20% increase in afferent responsiveness was detected.

#### Afferent recording experimental protocols

As described previously^16,17,35–37^, at the start of each afferent recording experiment, control bladder distensions were performed with an intravesical infusion of saline (NaCl, 0.9%) at a rate of 30 µL/min to a maximum pressure of 30 mmHg at 10 min intervals to assess the viability of the preparation and reproducibility of the intravesical pressure and neuronal responses to distension. The volume in the bladder was extrapolated from the known fill rate (30 µL/min) and the time taken (s) to reach maximum pressure (30 mmHg). Compliance was determined by plotting intravesical pressure against the calculated volume. After a stable baseline was maintained, the saline in the infusion pump was replaced by 5α-pregnan-3β-ol-20-one sulphate (Pg5α; 300 µM; Steraloids, #P3865-000) or 3-(2-chlorophenyl)-*N*-(4-chlorophenyl)-*N*,5-dimethyl-4-isoxazolecarboxamide (CCDC; 300 µM; Biovision, #1722-5, CAS: 1197300-24-5). Pg5α is a progesterone sulphate previously identified as an endogenous TGR5 agonist^28^, whilst CCDC is a potent synthetic TGR5 agonist reported to be TGR5-specific^38^. For both Pg5 α and CCDC, stock solutions were made to 10 mM with 1% DMSO. Stock solutions were diluted to final working concentrations in 0.9% saline. Control refers to 0.9% saline containing 0.03% DMSO. The single nerve bundle isolated and inserted into the glass electrode during the dissection process was contained within the recording electrode for the entire experiment, allowing afferent nerve responses to be compared in the same nerve fibres during intra-bladder incubation with saline and Pg5α (300 µM) or CCDC (300 µM).

### In vivo spinal dorsal horn activation

#### In vivo bladder distension

In vivo bladder infusion in anaesthetised mice were performed as previously described^16,37^. 12-week-old female mice were anaesthetised (isoflurane 2–4% in oxygen) and a catheter (PE 50 tubing) was inserted into the bladder via the urethra. Correct catheter placement was determined by inserting the catheter gently until meeting resistance (bladder dome) and receding slightly (2–3 mm) to avoid damaging the bladder wall. Urine was removed using a suction syringe and the catheter removed. A new catheter (PE 50 tubing), primed with either vehicle (saline; N = 5 mice) or CCDC (100 µM; N = 5 mice), was inserted and 100 µL of solution infused gently, to fill but not fully distend the bladder, and allowed to incubate for 5 min. The solution was then carefully removed from the bladder to ensure minimum liquid remained in the bladder. A third, dry catheter (PE 50 tubing) was inserted, and a small length of 4-0 perma-hand silk (Ethicon, #LA53G) was knotted over the urethral opening to fix the catheter into place and minimise loss of pressure. A 5-min stabilisation period was allowed between tying the catheter and performing the distensions to limit false-positive pERK signalling from the act of tying. A sphygmomanometer was attached between the catheter and a 10 mL air-filled suction syringe. 5 × 20-s bladder distensions were performed at 10-s intervals, at a holding pressure of 40 mmHg. Following the fifth 20-s distension, mice were administered an euthanasia agent overdose (intraperitoneal injection, 0.125 mL/250 g Lethabarb^®^; Virbac Australia) followed by rapid transcardial perfuse fixation.

#### Transcardial perfuse fixation and tissue dissection

As described previously^16,17,35–37^, following euthanasia overdose, the thoracic cavity was opened and 0.5 mL heparinised saline (50 IU in 5 mL; Pfizer) was injected into the left ventricle. A 22-gauge needle attached to tubing and a peristaltic perfusion pump was then inserted in or near the injection site in the left ventricle. The right atrium was cut open to allow for perfusate drainage. Warm phosphate buffer (0.1 M; 21.7 mM NaH_2_PO_4_ [Chem Supply, #SA061-500G], 81 mM Na_2_HPO_4_ [VWR Chemicals, #102494C], pH 7.2) was then perfused, followed by ice-cold 4% paraformaldehyde (PFA; Sigma-Aldrich, #158127) in 0.1 M phosphate buffer. After complete PFA perfusion, the lumbosacral spinal cord was removed and postfixed in 4% PFA in 0.1 M phosphate buffer at 4 °C for 18–20 h. Spinal cords were then cryoprotected in 30% sucrose/phosphate buffer (Sigma-Aldrich, #S9378) for two days at 4 °C, followed by a 48-h incubation at 4 °C in 50% optimal cutting temperature compound (OCT; VWR, #C361603E)/30% sucrose/phosphate buffer solution before freezing in 100% OCT using liquid nitrogen-cooled 2-methylbutane Sigma-Aldrich, #M32631). The frozen spinal cord segments were cryosectioned (10 µm thickness) and placed onto gelatin-coated slides for processing of immunofluorescence labelling. Sectioning was performed serially and distributed over 6 slides, which were used for immunofluorescence localisation of phosphorylated-MAP-kinase ERK 1/2 (pERK). The number of pERK-immunoreactive neurons were counted in spinal cord sections from L6-S1, with spinal segments determined ex vivo by the shape of the dorsal horn^39^.

#### Immunohistochemistry of phosphorylated-MAP-kinase ERK 1/2 within the spinal cord

The dorsal horn neurons activated by bladder distension were identified by immunofluorescent labelling for neuronal activation marker, phosphorylated-MAP-kinase ERK 1/2 (pERK), using a method described previously^16,36,37,40,41^. The details for the primary and secondary antisera can be found in Table 1. pERK activation was determined rather than c-Fos as ERK phosphorylation occurs within minutes of the peripheral stimuli and thus correlates better to the short-duration peripheral stimuli applied. Further, spinal neuronal activation in response to a range of peripheral stimuli is increasingly being identified via pERK activation quantification^36,37,41,42^. After air drying for one hour, excess OCT was removed from sections by washing with 0.2% Triton X-100 (Sigma-Aldrich, #T8787) in 0.1 M phosphate-buffered saline (PBS-T). Non-specific secondary antibody binding was blocked with 5% normal chicken serum diluted in 0.2% PBS-T. Tissue sections were then incubated with pERK primary antisera diluted in PBS-T at room temperature overnight (18 h). Sections were washed in PBS-T and incubated with secondary antibody conjugated to AlexaFluor 594 for 1 h at room temperature, followed by a PBS-T wash before mounting in ProLong Diamond Antifade (Life Technologies, #P-36962) and coverslipping. Negative controls were prepared as above with omission of the primary antibody. Slides were allowed to set for 24 h prior to visualisation.

**Table 1:** Primary and secondary antisera details.

Antigen	Species raised in	Manufacturer code	Manufacturer	Dilution	RIID	
**Primary**						
Phospho-p44/42 MAPK (Erk1/2) (Thr202/Tyr204) (D13.14.4E) XP(tm) mAb (pERK)	Rabbit	4370	Cell Signalling	1:100	AB_2315112	

Antigen	Species raised in	AF conjugate	Manufacturer code	Manufacturer	Dilution	RIID
**Secondary**						
Rabbit IgG H + L	Chicken	594	A21442	ThermoFisher	1:200	AB_141840

#### Microscopy

Fluorescence was visualised with an epifluorescence microscope (Olympus, Tokyo, Japan) using a 10 × objective and 3-s exposure. ImageJ software (NIH) was used for image analysis. Aside from making moderate adjustments for brightness and contrast, the images were not manipulated in any way.

#### Spinal cord pERK neuronal counts and analysis

Neuronal counts were analysed as described previously^16^ and utilized previously saved digital photomicrographs, with only neurons with intact nuclei counted. The number of pERK-immunoreactive (pERK-IR) neurons in a quadrant of the L6-S1 dorsal horn was obtained from a minimum of 6 sections/animal viewed at 10 × magnification. The mean number of pERK-IR neurons (± SEM) was compared between vehicle- and CCDC-treated mice.

### Retrograde tracing

As described previously^16^, small, aseptic, abdominal incision was made in anaesthetised (2–4% isoflurane in oxygen) mice. Cholera Toxin subunit B conjugated to AlexaFluor 488 (CTB-488, 0.5% diluted in 0.1 M phosphate-buffered saline [PBS] pH 7.4; ThermoFisher Scientific) was injected into the bladder wall at three sites (3 µL/injection) using a 5 µL Hamilton syringe attached to a 30-gauge needle^36,37^. The needle was inserted subserosally, parallel with the bladder muscle, to prevent injection of CTB into the bladder lumen. The abdominal incision was sutured closed and antibiotic (Amoxicillin; 50 mg/kg; Amoxil, AUSTR11137) and analgesic (Buprenorphine (Temvet); 0.1 mg/kg; Troy Laboratories Pty Ltd, APVMA #67612) given subcutaneously as mice regained consciousness. Mice were then individually housed and allowed to recover. 4 days post-surgery, mice were humanely killed for subsequent removal of the lumbosacral (LS; L5-S1) dorsal root ganglia (DRG) and isolation and culture of the neurons to visualise CTB-labelled bladder-innervating neurons within the DRG population.

### Cell culture of bladder-innervating DRG neurons

As described previously^16^, four days after retrograde tracing, mice were humanely euthanised via CO_2_ inhalation and lumbosacral (LS; L5-S1) dorsal root ganglia (DRG) were removed. DRG were digested in Hanks’ balanced salt solution (HBSS; pH 7.4; Life Technologies, #14170161) containing 4 mg/mL dispase (GIBCO, ThermoFisher Scientific, #17105041) and 3 mg/mL collagenase II (GIBCO, ThermoFisher Scientific, #17101015) at 37 °C for 30 min. The collagenase-dispase solution was aspirated and replaced with HBSS containing collagenase (3 mg/mL) only for 10 min at 37 °C, followed by washes in HBSS. DRG were then mechanically disrupted and cells dissociated in 600 µL complete DMEM (Dulbecco’s Modified Eagle Media [DMEM; GIBCO, ThermoFisher Scientific, #11995065]; 10% Foetal Calf Serum [Invitrogen, ThermoFisher Scientific, MA, USA]; 2 mM l-glutamine [GIBCO, ThermoFisher Scientific, #25030081], 100 µM MEM non-essential amino acids [GIBCO, ThermoFisher Scientific, #11140076], 100 mg/mL penicillin/streptomycin [GIBCO, ThermoFisher Scientific, #15070063], and 96 µg/L nerve growth factor-7S [Sigma, N0513-0.1MG]) via trituration through fire-polished Pasteur pipettes of descending diameter, and centrifuged at 50×*g* for 1 min^36,37,40,43^. Neurons were resuspended in complete DMEM (360 µL) and spot-plated onto 13 mm coverslips (30 µL/coverslip) coated with laminin (20 μg/mL; Sigma-Aldrich, #L2020) and poly-d-lysine (800 μg/mL; ThermoFisher Scientific) in a 12-well plate. Coverslips were incubated at 37 °C in 5% CO_2_ for 2–3 h to allow adherence of neurons before gently flooding each well with 1.7 mL complete DMEM. Cultured neurons were maintained in an incubator at 37 °C in 5% CO_2_ for 18–48 h for calcium imaging or cell picking for single-cell PCR as described below.

### Single-cell RT-PCR of individual bladder-innervating dorsal root ganglia neurons

Under continuous perfusion of sterile and RNA-/DNase-free PBS, single retrogradely traced bladder dorsal root ganglia (DRG) neurons were identified using a fluorescence microscope and collected into the end of a fine glass capillary using a micromanipulator^16,37^. The glass capillary was then broken into a sterile Eppendorf tube containing 10 µL of lysis buffer with DNAse (TaqMan Gene Expression Cells-to-CT Kit; Invitrogen, #4399002). For each coverslip of cultured DRG neurons, a bath control was also taken and analysed together with cells. Following lysis and termination of DNAse treatment, samples were immediately frozen on dry ice and stored at − 80 °C until cDNA synthesis was performed. RNA was reverse transcribed to cDNA using SuperScript™ IV VILO™ Master Mix (Invitrogen, #11766500) as per the manufacturer's instructions. cDNA was stored at − 20 °C for qRT-PCR. *Tubb3* (tubulin-3) expression served as a neuronal marker and positive control, and *Gfap* (glial fibrillary acidic protein) expression served as a glial cell marker for exclusion. Expression was considered positive if a complete curve was observed before 50 cycles^16^.

### Analysis of mRNA expression in whole LS DRG, isolated urothelial cells and bladder mucosal and detrusor layers

#### Tissue collection

As described previously^16^ 16- to 18-week-old mice were humanely euthanised via CO_2_ inhalation and the bladder and lumbosacral (L5-S1) dorsal root ganglia (DRG) removed. DRG were frozen in dry ice in pairs by spinal level (L5, L6, S1) and stored at – 80 °C for RNA extraction and quantitative reverse-transcription polymerase chain reaction (qRT-PCR). The bladder was dissected in sterile PBS with a single cut from the urethral opening along one side to the dome, stretched out, and pinned flat, urothelial side up. For RT-qPCR of the bladder layers, the mucosa and detrusor were gently peeled apart, frozen in dry ice, and stored at -80˚C for RNA extraction and qRT-PCR.

#### Isolation of mouse urothelial cells

For urothelial cell qRT-PCR, urothelial cells were isolated as performed previously^16,35,44,45^. Following stretching and pinning of the opened bladder as per bladder layer separation, the bladder was incubated with DMEM containing 2.5 mg/mL dispase, 10% Foetal Calf Serum, and 1 µM/mL HEPES (Sigma, #H3375, pH 7.0) at room temperature for 3 h. Cells were collected by gentle scraping of the urothelium with a blunt scalpel and dissociated in 0.025% trypsin EDTA (GIBCO, ThermoFisher Scientific, #25200072) at 37 °C in 5% CO_2_ for 15 min with gentle intermittent trituration^16^. The trypsin was deactivated by adding the cell suspension to DMEM containing 10% Foetal Calf Serum before centrifugation (15 min, 1500 rpm, 4 °C). Cells were then resuspended in keratinocyte serum-free media (KSFM; Invitrogen, #17005042). Cell count and viability were determined using the Countess Automated Cell Counter (Invitrogen, ThermoFisher Scientific). Cells were then pelleted, frozen in dry ice, and stored at − 80 °C for RNA extraction and qRT-PCR.

#### RNA extraction and cDNA synthesis

As described previously^16^ RNA was extracted using the PureLink RNA Micro kit (Invitrogen, Victoria, Australia, #12183-016; DRG pairs and isolated urothelial cells) or the PureLink RNA Mini kit (Invitrogen, #12183018A; bladder mucosa and detrusor) with DNAse treatment (Life Technologies, #12185-010) according to the manufacturer's instructions. A NanoDrop Lite spectrophotometer (Thermofisher Scientific) determined RNA quantity and purity. RNA was reverse transcribed to cDNA using SuperScript VILO Master Mix (Invitrogen, #11755250) as per the manufacturer's instructions. The cDNA was stored at − 20 °C until use.

#### Quantitative reverse-transcription polymerase chain reaction (qRT-PCR)

As described previously^16^ QRT-PCR was performed using Taqman Gene Expression Master Mix (Applied Biosystems, Victoria, Australia, #4369016) with commercially available hydrolysis probes (TaqMan; Life Technologies, see Table 2 for details) and RNAse-free water (AMBION, Victoria, Australia, #AM9916). For each reaction, 10 µL of MasterMix, 1 µL of TaqMan primer assay, 4 µL of water, and 5 µL of cDNA (1:2 dilution in RNA-free H_2_O) from each sample was tested in duplicate for each target. Reference genes *Actb* (DRG pairs) and *Gapdh* (mucosa, detrusor, and urothelial cells) were used as endogenous controls. Assays were run for 45 cycles on a 7500 Fast Real-Time PCR machine (Applied Biosystems), using 7500 Fast software, v2.0.6. mRNA quantities are expressed as 2^−ΔCt^ relative to reference gene. Using Prism 8 software (GraphPad, San Diego, CA), data were analysed by one-way analysis of variance (ANOVA) with Tukey’s multiple comparison test.

**Table 2:** Primers used for qRT-PCR receptor expression assays.

Gene Alias	Gene target	Assay ID
β-Actin (reference gene)	*Actb*	Mm00607939_s1
Gapdh (reference gene)	*Gapdh*	Mm99999915_g1
Tubulin-3 (neuronal marker)	*Tubb3*	Mm00727586_s1
Glial fibrillary acidic protein (glial marker)	*Gfap*	Mm01253033_m1
Takeda G-protein Receptor 5	*Gpbar1*	Mm04212121_s1
Transient receptor potential vanilloid 1	*Trpv1*	Mm01246300_m1
Transient receptor potential ankyrin 1	*Trpa1*	Mm01227437_m1

### Calcium imaging of cultured LS DRG neurons

As described previously^16,46^ cultured LS DRG neurons (18–48 h post-plating) were loaded with 2 μM Fura-2-acetoxymethyl ester (Fura-2; Invitrogen, ThermoFisher Scientific, #F1221) in pluronic F-127 (Invitrogen, ThermoFisher Scientific, #P3000MP) and incubated for 30 min at 37 °C before washing with HEPES buffer (10 mM HEPES sodium salt [4-(2-hydroxyethyl)piperazine-1-ethanesulfonic acid sodium salt; Sigma, #H7006-100G], 140 mM NaCl [Chem Supply, #SA046-3KG], 4 mM KCl [Chem Supply, #PA054-500G], 5 mM d-glucose anhydrous [Chem Supply, #GA018-500G], 2 mM CaCl_2_ [Scharlau, #CA01951000], and 2 mM MgCl_2_ [Sigma, #M8266-100G], pH 7.40) for 10 min and imaging at room temperature (23 °C). Fura-2 was excited at 340 and 380 nm and emissions were measured at 510 nm using a Nikon TE300 Eclipse microscope equipped with a Sutter DG-4/OF wavelength switcher, an Omega XF04 filter set for Fura-2, a Photonic Science ISIS-3 intensified CCD camera, and Universal Interface Card MetaFluor software. Retrogradely traced bladder-innervating DRG neurons were identified by the presence of the CTB-488 tracer, visible under excitation at 480 nm. 480 nm excitation was used for initial bladder neuron identification only; to prevent unnecessary photobleaching, images were not obtained at 480 nm during the experiment. Fluorescence images were obtained every 5 s leading up to and immediately following drug application, and every 20 s for washout period, using a 20× objective. Data were recorded and further analysed using MetaFluor software.

After an initial baseline reading to ensure cell fluorescence was stable, indicating healthy cells, DRG neurons were stimulated with CCDC (100 µM; Biovision, #1722-5, CAS: 1197300-24-5), and changes in intracellular calcium [Ca^2+^]_i_ were monitored in real time. For experiments assessing downstream TRP channel signalling, application and washout of CCDC was followed by application of TRPA1 agonist allyl isothiocyanate (AITC; 100 µM; Aldrich, #377430, CAS: 57-06-7) then TRPV1 agonist Capsaicin (50 nM; Sigma, #M2028, CAS: 404-86-4). After agonist additions, HEPES buffer containing high KCL (40 mM) was applied as a positive control to induce calcium overload driven by neuronal depolarisation. [Ca^2+^]_i_ is expressed as the ratio between the fluorescence signals at 340 and 380 nm. Baseline [Ca^2+^]_i_ was determined by averaging 8–12 fluorescence ratio readings immediately prior to stimulation. Maximum peak response was calculated by subtracting the relevant baseline score from the maximum fluorescence ratio measurement following compound application. Baseline fluorescence was normalised to a ratio of 1 for example traces.

### Data availability, experimental design, data analysis and statistics

The datasets used and/or analysed during the current study are available from the corresponding author on reasonable request. Data are presented as described previously^16^ with the mean ± SEM or the % of neurons or afferents or the maximum imp/s before and after. N indicates the number of animals, whilst n indicates the number of independent neurons or afferents. Statistical significance was reported at a level of *P < 0.05, **P < 0.01, ***P < 0.001, ****P < 0.0001. In some cases, # is used to indicate significance of at least *P* < 0.05 for multiple comparisons. Data were analysed using Prism 8 (GraphPad, San Diego, CA, USA), using one- or two-way analysis of variance (ANOVA) with Tukey’s or Sidak’s post hoc analyses dependent on data distribution, or student t-tests, for parametric data. Non-parametric data were analysed using Kruskal–Wallis test with Dunn’s multiple comparisons, Mann–Whitney analysis, or Wilcoxon matched-pairs signed rank test. Data were tested for Gaussian distribution to determine the correct statistical tests. The specific tests used to analyse each data set are indicated within individual figure legends.

### Ethics and animals

Animal studies were performed and reported in compliance with the ARRIVE guidelines. In this study, we used 12- to 18-week-old male and female C57BL/6J mice provided via an in-house breeding colony at the South Australian Health and Medical Research Institute (SAHMRI). Mice at SAHMRI were originally acquired from an in-house C57BL/6J breeding programme (JAX strain #000664; originally purchased from The Jackson Laboratory; breeding barn MP14; Bar Harbor, ME) and then bred within the specific and opportunistic pathogen-free animal care facility at SAHMRI. Some experiments utilised TRPV1 knockout (*trpv1*^*−/−*^), TRPA1 knockout (*trpa1*^*−/−*^), or TGR5 knockout (*Gpbar1*^*−/−*^) mouse strains with a C57BL/6J genetic background, also bred in-house at SAHMRI. *Trpv1*^*−/−*^ mice were originally acquired from an in-house breeding program at The Jackson Laboratory (JAX stock #003770; Bar Harbor, ME), *Trpa1*^*−/−*^ mice were gifted to us by the original developer, Prof David Corey (Harvard University, Cambridge, MA, USA), and *Gpbar1*^*−/−*^ mice were gifted to use by Prof Johan Auwerx and Prof Kristina Schoonjans, Ecole Polytechnique de Lausanne (Lausanne, Switzerland). *Gpbar1*^*−/−*^ mice were also crossed onto *Na*_*V*_*1.8*cre- line to generate a sensory neuron specific *Gpbar1*^−/−^ (*Na*_*V*_*1.8-Gpbar1*^*−/−*^). These genetically modified mouse lines were all then bred in-house at SAHMRI as per the wildtype mice. Na_V_1.8Cre-*Gpbar1*^*−/−*^ mice were gifted to us by Prof Nigel Bunnett (Monash University, Victoria, Australia), then housed at SAHMRI’s Preclinical, Imaging, and Research Laboratories (PIRL) facility for procedures.

All experiments were approved by and performed in accordance with the SAHMRI Animal Ethics Committee approvals (SAM195 and SAM190). Mice were group housed (5 mice per cage) within individual ventilated cages filled with chip coarse dust-free aspen bedding (PuraChips Aspen coarse 63L; Cat# ASPJMAEB-CA, Able Scientific, Australia). Cages were stored on individual ventilated cage racks within a temperature-controlled environment of 22 °C and a 12-h light/12-h dark cycle. Mice had free access to LabDiet JL Rat and Mouse/Auto6F chow (Speciality Feeds, Australia) and autoclaved reverse osmosis water. Sample sizes of each experiment are detailed in the corresponding figure legends.

## Results

### The TGR5 agonist Pg5α causes bladder afferent hypersensitivity

**Figure 1:**
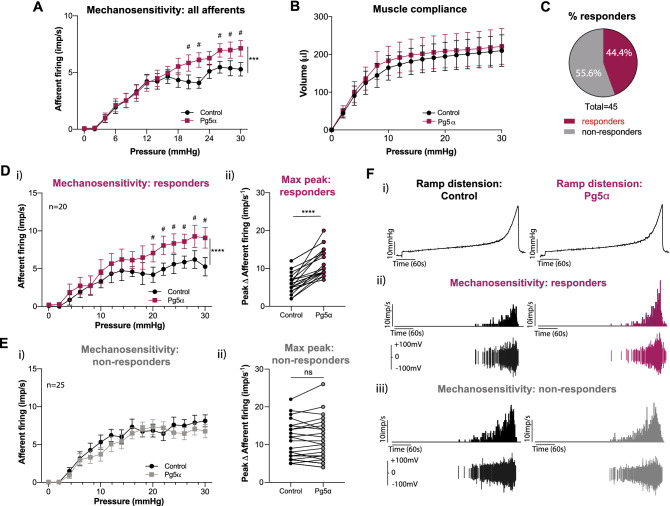
Pg5α enhances bladder afferent responses to mechanical stimuli ex vivo. (**A**) Whole nerve afferent firing was measured in response to graded bladder distension in an ex vivo preparation in the presence of saline (control) or progesterone sulphate ‘5α-pregnan-3β-ol-20-one sulphate’ (Pg5α; 300 µM). Application of Pg5α increased the overall bladder afferent firing rate (impulses per second; imp/s) in response to bladder distension compared to saline (n = 45 single units from N = 5 mice; ***P < 0.001 and ^#^*P* < 0.05 at individual distension pressures). (**B**) The intraluminal pressure measured as volume during bladder distension did not differ between saline and Pg5α (ns, *P* > 0.05), indicating no change in muscle compliance. (**C**) Of the 45 mechanosensitive bladder afferents recorded, 44.4% (20 out of 45) increased their responsiveness to distension following Pg5α (responders), whilst 55.6% (25 out of 45) were unchanged (non-responders). A single unit was deemed to be responsive if a greater than 20% increase in afferent responsiveness was detected. (**D**) Responding afferents had (i) significantly enhanced afferent firing rates at pressures of 20 mmHg and above (^#^*P* < 0.05) and (ii) a significantly higher maximum peak in afferent firing in the presence of Pg5α compared to saline (****P < 0.0001). (**E**) Non-responding mechanosensitive afferents had (**i**) no significant difference in afferent firing at any distension pressure and (**ii**) no significant difference in the maximum peak of afferent firing between distensions with saline or Pg5α (*ns*, *P* > 0.05). **F**) Representative examples demonstrating the (**i**) graded increase in pressure with volume over time in response to bladder distension, (**ii**) a Pg5α-responsive afferent during bladder distension in the presence of saline and Pg5α, and (**iii**) Pg5α non-responsive mechanosensitive afferent during bladder distension in the presence of saline and Pg5α. Data are represented as mean ± SEM (**A**,**B**,**Di**,**Ei**), or percentage of afferents (**C**), or maximum imp/s before and after Pg5α (**Dii**,**Eii**). ‘Responders’ were defined by a greater than 25% increase in afferent firing (**C**,**D**,**E**,**F**). P values are based on two-way ANOVA (**A**,**B**,**Di**,**Ei**, with subsequent Sidak’s post hoc test significance at individual data points indicated (**A**,**Di**, # represents significance of at least *P* < 0.05), or Wilcoxon matched-pairs signed rank test (**Dii**,**Eii**).

To determine if activation of the TGR5 receptor could influence bladder sensation we determined the impact of peripheral TGR5 activation in an ex vivo bladder afferent preparation. Intravesical application of Pg5α, a progesterone sulphate previously identified as an endogenous TGR5 agonist^28^, resulted in a significant increase in bladder afferent firing in response to graded bladder distension compared to saline (Fig. 1A), indicating mechanical hypersensitivity following TGR5 activation. No change was observed in bladder compliance (pressure/volume relationship) between saline and Pg5α experiments (Fig. 1B), indicating that afferent hypersensitivity was not caused by changes in detrusor muscle tone.

Single-unit analysis of the bladder afferent nerve fibres revealed that 44.4% (20/45) of the mechanosensitive bladder afferents demonstrated mechanical hypersensitivity following instillation of Pg5α (‘responders’), whereas 55.6% (25/45) of mechanosensitive afferents were unresponsive to Pg5α (‘non-responders’) (Fig. 1C). Pg5α-responsive afferents showed a significantly increased afferent firing rate at pressures of 20 mmHg and above (Fig. 1Di) and a significant increase in the maximum peak of afferent firing in response to distension following Pg5α instillation (Fig. 1Dii). In comparison, no change in afferent firing was observed between saline and Pg5α experiments for non-responding afferents, neither at any particular distension pressure (Fig. 1Ei) nor in the maximum peak response (Fig. 1Eii). These data demonstrate that the progesterone metabolite Pg5α causes mechanical hypersensitivity of a sub-population of bladder afferents in response to graded bladder distension (Fig. 1F).

### The synthetic TGR5 agonist CCDC replicates Pg5α-induced increases in bladder afferent mechanosensitivity

**Figure 2:**
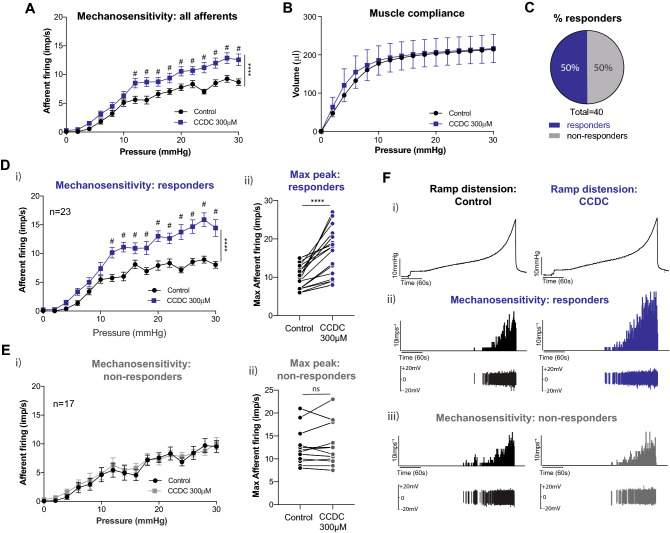
The synthetic TGR5 agonist CCDC enhances bladder afferent mechanosensitivity. (**A**) Afferent firing was recorded in response to graded bladder distension in an ex vivo preparation, comparing responses in the presence of saline (control) or the synthetic TGR5 agonist CCDC. Application of CCDC (300 µM) caused a significant increase in the overall afferent firing rate (impulses per second; imp/s) at distension pressures at and above 12 mmHg (n = 40 single units from N = 4 mice; ****P < 0.0001, and ^#^*P* < 0.05 at individual distension pressures). (**B**) No differences were observed in the pressure to volume relationship between saline and CCDC (ns, *P* > 0.05). (**C**) Of the 40 mechanosensitive bladder afferents recorded, 50% (20 out of 40) displayed enhanced responses to distension after CCDC (responders), whilst 50% (20 out of 40) were unchanged. (**D**) CCDC-responsive afferents (n = 20) demonstrated a significant increase in mechanosensitivity following CCDC application. (**i**) Responders showed significantly increased mechanosensitivity to distension at pressures at and above 12 mmHg (**** *P* < 0.0001). (**ii**) The maximum peak of afferent firing with CCDC was significantly higher compared with saline (****P < 0.0001). (**E**) CCDC-unresponsive mechanosensitive afferents (non-responders; n = 20) showed no difference in mechanosensitivity between saline and CCDC, with (**i**) no difference in imp/s at any distension pressure (ns, *P* > 0.05) and (**ii**) no change observed in the maximum imp/s to bladder distension (ns, *P* > 0.05). (**F**) (**i**) Example experimental traces showing the graded increase in pressure that occurs with increasing volume over time with saline, followed by CCDC. (**ii**) Graded increases in afferent firing in response to bladder distension, with a visible increase in imp/s following administration of CCDC (100 µM). (**iii**) Graded increase in afferent firing in response to bladder distension, with no change in response between saline and CCDC administrations. Data are represented as mean ± SEM (**A**,**B**,**Di**,**Ei**), percentage of afferents (**C**), or maximum imp/s (**Dii**) and **Eii**). ‘Responders’ were defined by a greater than 25% increase in afferent firing (**C**,**D**,**E**). P values above are based on two-way ANOVA (**A**,**B**,**Di**,**Ei**), with subsequent Sidak’s post hoc test significance at individual data points indicated (**A**,**Di**, # represents significance of at least *P* < 0.05), or Wilcoxon matched-pairs signed rank test (**Dii**,**Eii**).

We next aimed to further define and confirm the specific involvement of the TGR5 receptor in mediating bladder afferent mechanosensitivity. To this end, we used a potent, synthetic TGR5 agonist, 3-(2-chlorophenyl)-*N*-(4-chlorophenyl)-*N*,5-dimethyl-4-isoxazolecarboxamide (CCDC), reported to be TGR5-specific^38^ to confirm the results obtained with Pg5α. Similar to Pg5α, on applying CCDC to the ex vivo bladder afferent preparation we observed an increase in afferent mechanosensitivity to bladder distension compared to saline (Fig. 2A). No change to muscle compliance was observed between saline and CCDC intravesical instillation (Fig. 2B), demonstrating that the effect of CCDC on mechanosensitivity is a direct afferent effect and not secondary to muscular changes. Single unit analysis of the mechanosensitive bladder afferents showed 50% (20/40) of afferents responded to CCDC with mechanical hypersensitivity to bladder distension compared to saline (‘responders’), while 50% (20/40) showed no change in mechanosensitivity following CCDC instillation (‘non-responders’) compared to saline (Fig. 2C). CCDC-responsiveness was indicated by a mechanical hypersensitivity compared to saline at pressures of 10 mmHg and above (Fig. 2Di) and by a significant increase in the maximum peak of afferent firing (Fig. 2Dii) following CCDC instillation compared to saline. No changes in afferent firing at any individual pressures (Fig. 2Ei) or at maximum peak (Fig. 2Eii) were observed in the non-responding afferents. These data demonstrate that CCDC causes mechanical hypersensitivity of a sub-population of bladder afferents (Fig. 2F).

### CCDC-induced bladder afferent mechanical hypersensitivity is reduced in ***Gpbar1***^***−/−***^ mice

**Figure 3:**
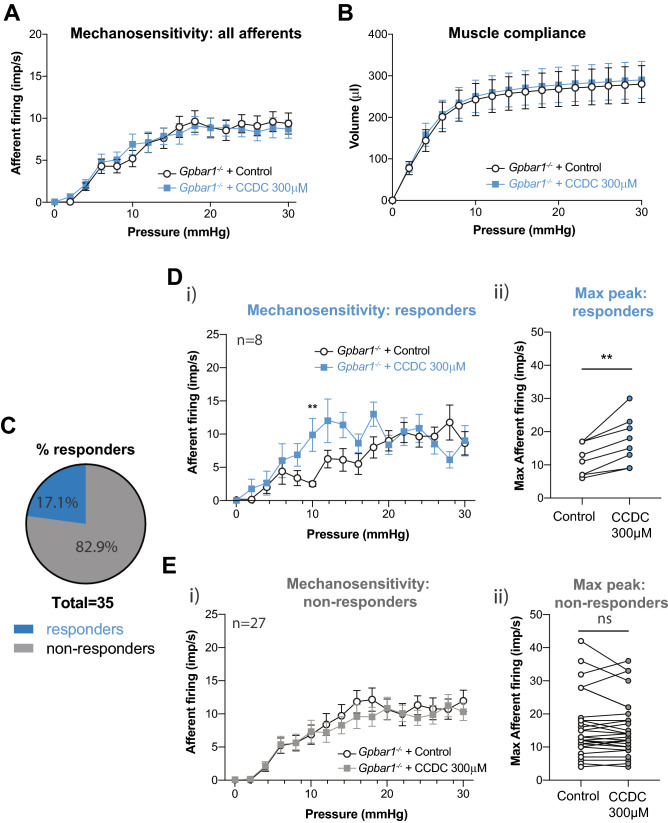
CCDC-induced hypersensitivity to distension is abolished in *Gpbar1 (TGR5)*^*−/−*^ mice. (**A**) Bladder distensions were performed with saline (control) and CCDC (100 µM) *in* ex vivo preparations from *Gpbar1*^*−/−*^ mice. No difference in afferent firing (impulses per second; imp/s) was observed following administration of CCDC compared to saline in the *Gpbar1*^*−/−*^ mice (n = 35 single units from N = 4; ns, *P* > 0.05). (**B**) The pressure to volume relationship during bladder distension is unchanged following CCDC administration compared to saline, indicating no change in muscle compliance in preparations from *Gpbar1*^*−/−*^ mice (ns, *P* > 0.05). (**C**) Whilst the majority of afferents (82.9%) reflected the overall results with no change in afferent firing (29 out of 35; non-responders), 6 out of 35 (17.1%) afferents displayed enhanced responses to distension following CCDC with an increase in mechanosensitivity. (**D**) In CCDC-responsive afferents, (**i**) a significant increase in imp/s was observed following CCDC administration overall (**P < 0.01) and specifically at a distension pressure of 10 mmHg (**P < 0.01). (**ii**) the maximum imp/s following CCDC administration was significantly increased compared to saline in responders (**P < 0.01). (**E**) In non-responding afferents, (**i**) no change in imp/s at any distension pressure was observed in non-responders between CCDC and saline distensions (ns, *P* > 0.05), whilst (**ii**) no significant difference was observed between the maximum imp/s of non-responders following administration of CCDC compared to saline (ns, *P* > 0.05). P values above are based on two-way ANOVA (**A**,**B**,**Di**,**Ei**), with subsequent Sidak’s post hoc test significance at individual data points indicated on (**Di**), Wilcoxon matched-pairs signed rank test (**Dii**,**Eii**).

To confirm that the CCDC-evoked mechanical hypersensitivity in ex vivo bladder afferents we observed were mediated by TGR5, we determined the effect of CCDC on bladder afferents in a *Gpbar1*^*−/−*^ mouse model. In *Gpbar1*^*−/−*^ mice, the mechanical hypersensitivity to distension following CCDC compared to saline was completely abolished (Fig. 3A). As in wildtype mice there was no change in muscle compliance between CCDC and saline experiments (Fig. 3B), confirming that the absence of altered sensitivity was not caused by changes in bladder muscle function. Interestingly, single unit analysis of the afferents showed that while the majority of afferents (82.9%; 29/35) reflected the overall lack of sensitivity to CCDC, mechanical hypersensitivity was observed in 17.1% (6/35) of mechanosensitive afferent fibres (Fig. 3C). These CCDC-responsive neurons demonstrated mechanical hypersensitivity specifically at 10 mmHg (Fig. 3Di) and an increase in the maximum peak of afferent firing (Fig. 3Dii). In line with the overall bladder afferent data, the vast majority of mechanosensitive single units (82.9%; 29/35) showed no difference in mechanosensitivity following CCDC instillation compared to saline (Fig. 3Ei-ii).

### In vivo intravesical CCDC in wild-type mice increases the number of activated dorsal horn neurons to bladder distension, an effect not apparent in* Gpbar1*^−/−^ mice

**Figure 4:**
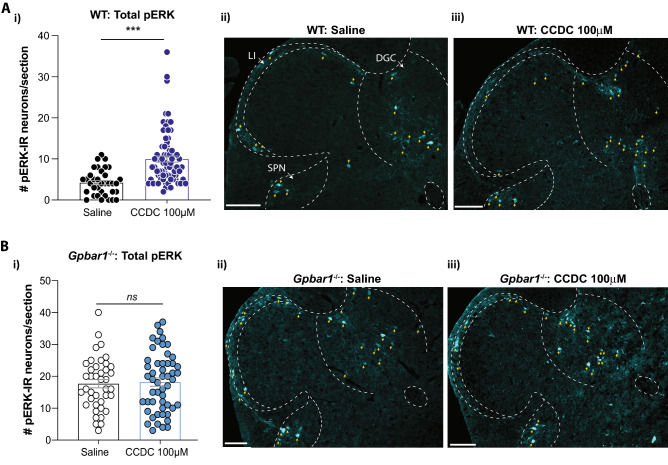
Intravesical bladder CCDC administration increases spinal dorsal horn pERK immunoreactivity in response to bladder distension. (**A**) (**i**) In vivo bladder distensions in wildtype mice following intravesical saline (control) or CCDC (100 µM) administration. Group data showing the number of pERK-immunoreactive (pERK-IR) dorsal horn neurons in 10 µm sections of the lumbosacral spinal cord. Compared to saline (N = 4), the number of pERK-IR neurons in the LS dorsal horn per section was significantly greater in response to bladder distension following intravesical administration of CCDC (N = 6; ***P < 0.001). (**ii–iii**) Images are representative dorsal horn sections from the S1 region from (**ii**) saline-treated and (**iii**) CCDC-treated wildtype mice, with dashed lines to delineate the superficial dorsal horn (SDH) and dorsal grey commissure (DGC), and yellow arrows pointing to pERK-IR neurons. (**B**) (**i**) In *Gpbar1*^−/−^ mice there was no difference observed in the number of pERK-IR neurons in the LS dorsal horn per section following bladder distension following intravesical administration of either saline (N = 3) or CCDC (N = 4; ns, *P* > 0.05). Images are representative dorsal horn sections from the S1 regions from (**ii**) saline-treated and (**iii**) CCDC-treated *Gpbar1*^*−/−*^ mice, with dashed lines to delineate the superficial dorsal horn (SDH) and dorsal grey commissure (DGC), and yellow arrows pointing to pERK-IR neurons. Data are represented as mean ± SEM, with each dot representing the total number of pERK-IR neurons within a single dorsal horn quadrant from one section. *P* values were determined by Mann–Whitney non-parametric test (**Ai**,**Bi**). Scale bar 100 μm throughout. *CC* central canal, *SPN* sacral parasympathetic nucleus, *LSN* lateral spinal nucleus.

Our results thus far have established a role for TGR5 agonists in peripheral bladder afferent hypersensitivity. To determine whether this mechanical hypersensitivity is conveyed to the central nervous system, we assessed dorsal horn neuronal activation in response to in vivo bladder distension at 40 mmHg induced pERK-immunoreactivity in lumbosacral (LS) dorsal horn neurons (Fig. 4A). We observed LS dorsal horn pERK-immunoreactive (-IR) neurons in response to saline bladder distension at 40 mmHg in wild-type mice (Fig. 4Ai). The pERK-IR neurons were identified predominantly in the regions of the dorsal grey commissure and the superficial dorsal horn, with some pERK-IR neurons in the lateral spinal nuclei and the sacral parasympathetic nucleus in sacral spinal segments (Fig. 4Aii). Following intra-bladder CCDC application we observed a significant increase in the number of pERK-IR neurons within LS dorsal horn which were distributed throughout the same regions of the dorsal horn as seen in the saline experiments (Fig. 4Ai, Aiii). As these regions are known to play a role in bladder afferent input, integration, and projection of autonomic and nociceptive visceral signalling^36,47^, an increase in pERK-IR neurons indicates an increase in central sensitivity to bladder distension and hence projection of the peripheral mechanosensitivity observed ex vivo in wild-type mice.

LS spinal dorsal horn neuronal pERK-IR induced by in vivo bladder distensions following intravesical incubation with CCDC or saline was also determined in *Gpbar1*^*−/−*^ mice. The CCDC-induced enhanced spinal dorsal horn neuronal response to bladder distension was abolished in the *Gpbar1*^*−/−*^ model, with no difference in the number of LS dorsal horn pERK-IR neurons between CCDC and saline in these mice (Fig. 4Bi). The pERK-IR neurons activated by bladder distension following intravesical instillation of both saline and CCDC were observed in the same dorsal horn regions as seen in the wildtype, suggesting no differences in spinal bladder afferent signalling pathways in the *Gpbar1*^*−/−*^ mice (Fig. 4Bii,Biii). Taken together, these results demonstrate that activation of the TGR5 receptor is able to mediate an increase in peripheral and spinal sensitivity to bladder distension indicative of sensory neuron activation.

### *Gpbar1* (TGR5) mRNA is expressed in the bladder and bladder innervating DRG neurons

**Figure 5:**
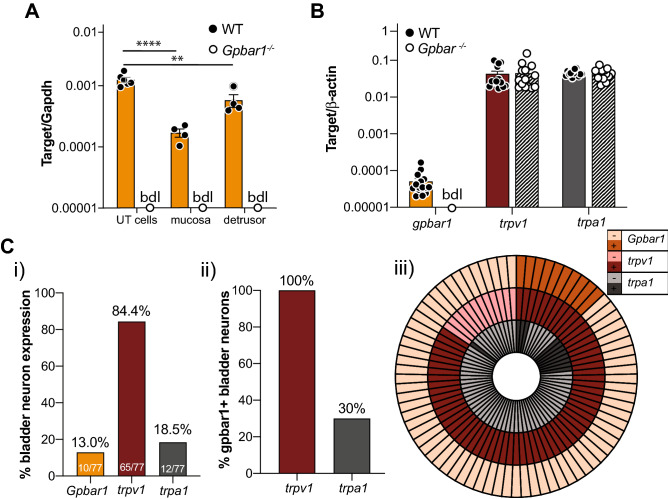
Relative mRNA expression in bladder tissue, lumbosacral DRG and isolated bladder-innervating DRG neurons. (**A**) qRT-PCR was used to determine the relative abundance of the TGR5 gene *Gpbar1* in urothelial cells, mucosal urothelium, and detrusor smooth muscle of the bladder of wild-type and *Gpbar1*^−/−^ mice. *Gpbar1* was expressed in all layers of the wildtype bladder, with relative abundance in the isolated urothelial cells (UT cells; N = 6 mice) significantly higher than in the mucosa (N = 4 mice; ****P < 0.0001) and detrusor (N = 4 mice; **P < 0.01). No significant difference was observed between mucosa and detrusor layers (ns, *P* = 0.0833). *Gpbar1* mRNA expression was below detectable levels (bdl) in all tissues tested from *Gpbar1*^−/−^ mice. (**B**) Relative mRNA expression of *Gpbar1*, *trpv1* and *trpa1* in lumbosacral (LS) dorsal root ganglia (DRG) from wildtype and *Gpbar1*^*−/−*^ mice. *Gpbar1* is expressed in LS DRG from WT mice. *Gpbar1* levels were below the detectable limit (bdl) in *Gpbar1*^*−/−*^ mice. No differences were observed between wildtype and *Gpbar1*^*−/−*^ mice for the highly expressed genes *trpv1* or *trpa1*. (**C**) (**i**) *Gpbar1*, *trpv1* and *trpa1* mRNA expression in isolated, retrogradely traced bladder-innervating DRG neurons was determined with single cell RT-PCR. 13% of bladder neurons (10 out of 77 neurons, N = 3) expressed *Gpbar1*, while 84.4% (65 out of 77 neurons) expressed *trpv1* and 18.5% (12 out of 77 neurons) expressed *trpa1*. (**ii**) Of the 10 bladder-innervating DRG neurons expressing *Gpbar1*, 100% (10 out of 10) co-expressed *trpv1*, while 30% (3 out of 10) co-expressed *trpa1*. (**iii**) Polar plot depicting the expression profile (dark shading: expressing target; light shading: not expressing target) of each bladder-innervating DRG neuron with respect to *Gpbar1* (outer ring), *Trpv1* (middle ring) and *Trpa1* (inner ring). Data are represented as mean ± SEM (2^−ΔCt^) on a log10 scale (**A**,**B**), or percentage of neurons (**Ci**,**Cii**). *P* values above are based on one-way ANOVA with Tukey’s multiple comparison test, with *Gpbar1*^−/−^ not included in the analysis.

To begin unravelling the mechanisms through which TGR5 agonists CCDC and Pg5α exert mechanical hypersensitivity of bladder afferents we determined the relative mRNA expression of the TGR5 gene *Gpbar1* within the bladder and bladder-innervating sensory neurons. *Gpbar1* mRNA expression was identified throughout the bladder wall; in isolated urothelial cells, the mucosa, and the detrusor smooth muscle (Fig. 5A), as well as in isolated LS (L5-S1) DRG (Fig. 5B). *Gpbar1* expression was absent in all tissues from *Gpbar1*^*−/−*^ mice (Fig. 5A,B), whilst the relative abundance of either Transient receptor potential (TRP) vanilloid 1 (*trpv1*) or ankyrin 1 (*trpa1*) observed in LS DRG from *Gpbar1*^*−/−*^ mice was unchanged compared to wildtype (Fig. 5B). TRP channels are common downstream targets for GPCR signalling cascades, with previous studies identifying downstream coupling of irritant-sensing receptors with TRPV1 and TRPA1^16,24,26,48,49^. To assess potential TRP channel coupling with TGR5 in bladder neurons, we used single cell RT-PCR to determine the co-expression of *Gpbar1* with *trpv1* and *trpa1.* Single cell RT-PCR identified *Gpbar1* expression in 13.0% (10/77) of retrogradely traced bladder afferent neurons (Fig. 5C). 84.4% (65/77) and 18.5% (12/77) of bladder-innervating DRG expressed *trpv1* and *trpa1* mRNA, respectively (Fig. 5C). Of the bladder DRG neurons expressing *Gpbar1* mRNA, 100% co-expressed *trpv1*, and only 30% co-expressed *trpa1* (Fig. 5Cii,Ciii), despite previous functional data in other DRG neurons showing distinct coupling of TGR5 with TRPA1^24^.

### In vitro application of CCDC to bladder-innervating DRG neurons increases intracellular Ca^2+^

**Figure 6:**
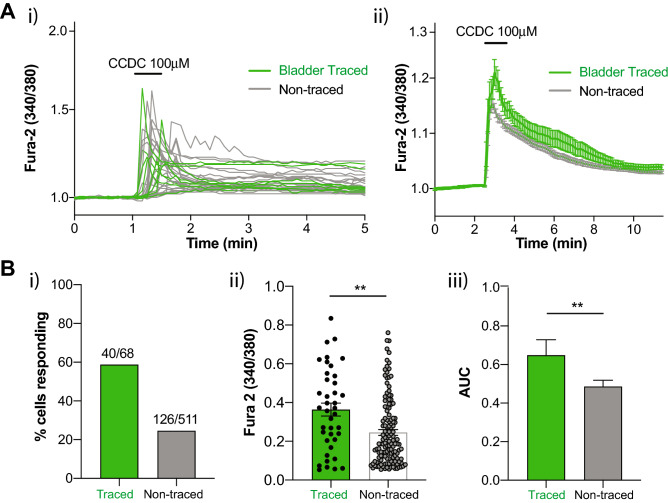
In vitro activation of TGR5 in primary DRG neurons results in increased intracellular Ca^2+^. (**A**) Live cell calcium imaging was performed on retrogradely traced, isolated LS DRG neurons from wild-type mice to measure intracellular Ca^2+^ changes in response to direct CCDC (100 µM) application. (**i**) An example experiment showing bladder traced (green) and non-traced (grey) DRG neurons from a single coverslip, with each line representing a single neuron over time and an increase in the Fura-2 fluorescence ratio (340/380) indicating an increase in intracellular Ca^2+^ from baseline normalised to a ratio of 1. (**ii**) Mean ± SEM of intracellular Ca^2+^ responses to CCDC (100 μM) in traced (n = 40) and non-traced (n = 126) neurons. (**B**) (**i**) 58.82% of bladder traced neurons (40 out of 68; N = 7) responded to CCDC application with an increase in intracellular Ca^2+^, compared to only 24.65% of non-traced neurons (126 out of 511). The response to CCDC for traced and non-traced neurons showed (**ii**) a significantly higher maximum peak (****P < 0.0001) and (**iii**) a significantly larger area under the curve (AUC, **P < 0.01) in bladder-innervating DRG neurons compared to non-traced DRG neurons.

To gain insights into the mechanism of action of neuronal TGR5 in mediating bladder afferent sensitivity, we performed live cell calcium imaging in LS DRG neurons isolated from retrogradely bladder-traced mice. Following direct application of CCDC to the neurons, a significant increase in intracellular Ca^2+^, indicated by an increase in the Fura-2 fluorescence ratio (Fig. 6Ai,Aii), was observed in 58.82% (40/68) of bladder-traced DRG neurons (Fig. 6Bi). Interestingly, the percentage of bladder-traced neurons responding to CCDC was substantially higher than the percentage on non-traced neurons, of which only 24.65% (126/511) responded (Fig. 6Bi). The average magnitude of CCDC-induced increases in intracellular Ca^2+^ was also greater in bladder-traced compared to non-traced neurons, as indicated by a significantly higher maximum peak value of Fura-2 fluorescence and area under the curve of response (Fig. 6Bii,Biii). These results demonstrate the TGR5 agonist CCDC is able to cause direct activation of LS DRG neurons and indicate the possibility that bladder-innervating DRG neurons are tuned to evoke greater responses to TGR5 agonists.

### In vitro CCDC-induced increases in intracellular Ca^2+^ in bladder-innervating DRG neurons are partially mediated by TRPV1

**Figure 7:**
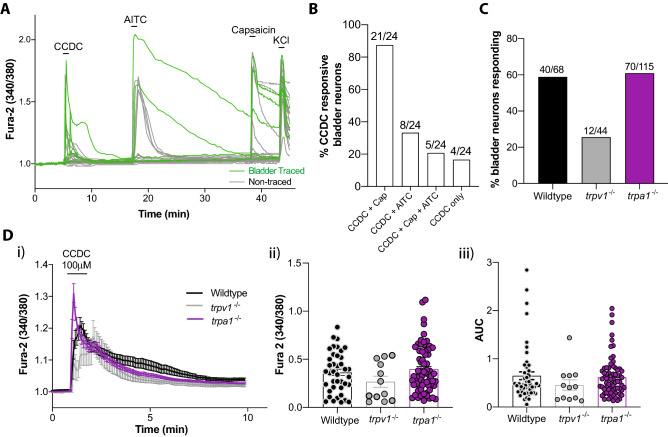
Intracellular calcium responses to CCDC in *trpv1*^−/−^ and *trpa1*^−/−^ mice. (**A**) Live cell calcium imaging was performed on isolated, bladder-traced DRG neurons from WT, *trpv1*^*−/−*^ and *trpa1*^*−/−*^ mice to determine changes in intracellular Ca^2+^ in response to direct application of CCDC. An example experiment showing bladder traced (green) and non-traced (grey) neurons from a single coverslip, with each line representing a single neuron over time and an increase in the Fura-2 fluorescence ratio (340/380) indicating an increase in intracellular Ca^2+^, from baseline normalised to a ratio of 1. The TRPA1 agonist AITC (100 µM) and TRPV1 agonist capsaicin (50 nM) were applied in succession following CCDC (100 µM) and washout periods to determine co-activation of neurons. KCl was added at the end of each experiment as a positive neuronal control. (**B**) The percentage of neurons co-responding with CCDC to each compound. Of the CCDC-responsive neurons 87.5% (21/24) also responded to capsaicin, whilst 33.3% (8/24) of CCDC-responsive bladder-traced neurons also responded to AITC. Only 16.7% (4/24) of CCDC-responsive bladder-traced neurons were unresponsive to both AITC and capsaicin. (**C**) The percentage of bladder-traced neurons responding to CCDC in WT, *trpv1*^*−/−*^ and *trpa1*^*−/−*^ mice. Compared to 58.8% observed in wildtype neurons, only 27.3% of *trpv1*^*−/−*^ bladder-traced neurons (12 out of 44; N = 4) responded to CCDC, whilst 60.9% of bladder-traced neurons from *trpa1*^*−/−*^ mice responded to CCDC (70 out of 115; N = 5). (**D**) (**i**) An average response curve for each population was determined from the CCDC-responsive bladder-innervating DRG neurons, to visually compare the neuronal responses between wildtype (black), *trpv1*^*−/−*^ (grey), and *trpa1*^*−/−*^ (purple) bladder neurons. (**ii**) maximum peak fluorescence and (**iii**) area under the curve (AUC) of fluorescence were compared across neurons from wildtype (black), *trpv1*^*−/−*^ (grey), and *trpa1*^*−/−*^ (purple) mice. No significant differences between either *trpv1*^*−/−*^ or *trpa1*^*−/−*^ neurons and wild-type neurons were observed. Data are represented as percentage of neurons (**B**,**C**) or mean ± SEM (**Di**,**Dii**,**Diii**). *P* values were determined using Kruskal–Wallis non-parametric ANOVA tests with subsequent Dunn’s post hoc multiple comparisons tests.

Coupling between TGR5 and TRPA1 has been previously demonstrated in cutaneous^24^ and colon^26^ innervating DRG neurons. Here our single-cell mRNA expression data from bladder-innervating DRG neurons demonstrated 100% co-expression of *trpv1* with *Gpbar1* compared to only 30% co-expression of *trpa1* with *Gpbar1* (Fig. 5C), suggesting a distinct intracellular signalling pathway in bladder-innervating DRG neurons. To functionally explore potential coupling between TGR5 and TRP channels in bladder afferent DRG neurons in vitro, we determined co-activation of TGR5, TRPA1, and TRPV1 in individual neurons with successive administration of CCDC, AITC (TRPA1 agonist), and capsaicin (TRPV1 agonist) using live cell calcium imaging (Fig. 7A,B). In line with our single-cell co-expression data, 87.5% (21/24) of bladder traced neurons that responded to CCDC also responded to capsaicin, whilst 33.3% (8/24) of CCDC-responsive bladder-traced neurons also responded to AITC (Fig. 7B). Only 16.7% (4/24) of CCDC-responsive bladder-traced neurons were unresponsive to both AITC and capsaicin (Fig. 7B).

To further explore the potential functional relationships between TGR5 and TRP channels TRPV1 and TRPA1, we measured bladder-traced DRG neuronal responses to CCDC in vitro using *trpv1*^*−/−*^ and *trpa1*^*−/−*^ mice. The percentage of bladder-traced DRG neurons responding to CCDC was reduced by approximately 50% in *trpv1*^*−/−*^ mice (27.3%) compared to wildtype mice (58.8%), with no obvious change observed in the *trpa1*^*−/−*^ mice (60.9%) compared to wildtype mice (Fig. 7C). The profile and magnitude of CCDC responses between wildtype and *trpv1*^*−/−*^ and *trpa1*^*−/−*^ mice was also examined (Fig. 7Di–iii). Whilst the few *trpv1*^*−/−*^ neurons that responded to CCDC show a trend towards reduced response amplitudes, there was no statistical differences in the average maximum peak of Fura-2 fluorescence (Fig. 7Dii) and area under the curve (Fig. 7Diii) of the CCDC responses. Overall, these data implicate an interaction between TRPV1 and TGR5 in subsets of bladder-innervating DRG neurons. However, as CCDC responses are also observed in some *trpv1*^−/−^ DRG neurons, this suggests interactions with other channels also occur.

### In vitro CCDC-induced increases in intracellular Ca^2+^ in bladder-innervating DRG neurons remain in ***Gpbar1***^−/−^ mice

**Figure 8:**
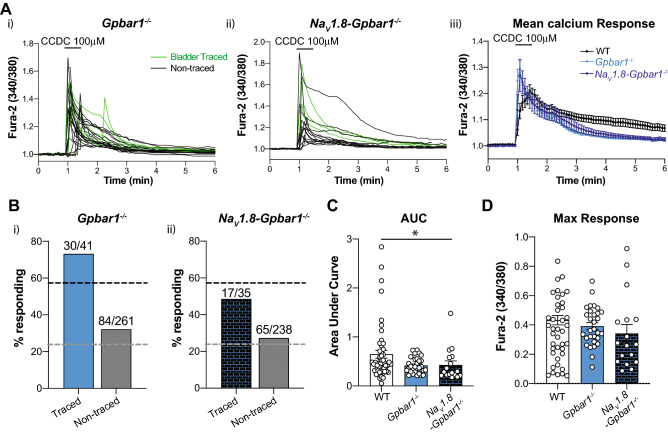
Intracellular calcium responses to CCDC in neuronal cultures from *Gpbar1*^−/−^ and *Na*_*V*_*1.8-Gpbar1*^−/−^ mice. (**A**) Intracellular calcium changes were measured in bladder-traced (green) and untraced (black) lumbosacral dorsal root ganglia (LS DRG) neurons from *Gpbar1*^*−/−*^ and *Na*_*V*_*1.8*-*Gpbar1*^*−/−*^ mice in response to CCDC (100 µM). Example experiments from (**i**) *Gpbar1*^*−/−*^ and (**ii**) *Na*_*V*_*1.8*-*Gpbar1*^*−/−*^ mice showing bladder traced (green) and non-traced (black) neurons from a single coverslip responding to CCDC (100 μM). Each line represents a single neuron and an increase in the Fura-2 fluorescence ratio (340/380) indicates an increase in intracellular Ca^2+^. (**iii**) Mean ±  SEM of intracellular Ca^2+^ responses to CCDC (100 μM) in bladder-innervating neurons from Wildtype, *Gpbar1*^*−/−*^ and *Na*_*V*_*1.8-Gpbar1*^*−/−*^ mice. (**B**) The percentage (%) of neurons responding to 100 µM CCDC in *Gpbar1*^*−/−*^ and *Na*_*V*_*1.8*-*Gpbar1*^*−/−*^ mice. (**i**) 73.2% (30 out of 41) of bladder-traced *Gpbar1*^*−/−*^ neurons, and (**ii**) 48.6% (17 out of 35) of bladder-traced *Na*_*V*_*1.8*-*Gpbar1*^*−/−*^ neurons responded to CCDC. Dashed black and grey lines represent % of bladder-innervating neurons and untraced neurons from wildtype mice responding to CCDC (100 µM). (**C**) The average intracellular calcium change over time (AUC) in response to 100 µM CCDC was determined for *Gpbar1*^*−/−*^ (light blue) and *Na*_*V*_*1.8*-*Gpbar1*^*−/−*^ (dark blue) bladder-traced LS DRG neurons and compared to wildtype (white). No statistical difference in the area under the curve (AUC) was observed between wildtype and *Gpbar1*^*−/−*^ responses (ns, *P* > 0.05), whereas *Na*_*V*_*1.8*-*Gpbar1*^*−/−*^ bladder-traced responses had a significantly reduced AUC compared to wildtype (*P < 0.05). No significant difference was observed in the AUC between *Gpbar1*^*−/−*^ and *Na*_*V*_*1.8*-*Gpbar1*^*−/−*^ responses. (**D**) Maximum fluorescent responses to CCDC (100 µM) were not significantly different between wildtype, *Gpbar1*^*−/−*^ and *Na*_*V*_*1.8*-*Gpbar1*^*−/−*^ bladder-traced neurons (ns, *P* > 0.05). Data are represented as percentage of cells (**B**) or mean ± SEM (**C**,**D**) with individual data points in (**C**,**D**) representing values from individual cells. *P* values above were determined by Kruskal–Wallis non-parametric analysis of variance with Dunn’s multiple comparisons tests (**C**,**D**).

To confirm the in-vitro effects of CCDC on isolated bladder-innervating DRG neurons were due to activation of the TGR5 receptor we examined CCDC responses firstly in cells isolated from *Gpbar1*^*−/−*^ mice and subsequently in *Na*_*V*_*1.8Cre-Gpbar1*^−/−^ mice. To our surprise, responses to CCDC remained in neurons from these mice (Fig. 8). The percentage of cells responding to CCDC from *Gpbar1*^*−/−*^ and *Na*_*V*_*1.8Cre-Gpbar1*^*−/−*^ mice was roughly equivalent to that from wild-type mice (Fig. 8Ai–iii,Bi–ii). The total fluorescent response to CCDC, depicted as the area under the response curve (AUC) was significantly reduced in cells from *Na*_*V*_*1.8Cre-Gpbar1*^*−/−*^ (neuronal knockout of *Gpbar1*) but not *Gpbar1*^*−/−*^ (global knockout of *Gpbar1*) mice (Fig. 8Aiii,C), however, a robust response still remained. No significant differences in the maximum peak of Fura-2 fluorescence in response to CCDC were observed in cells from wildtype, *Gpbar1*^*−/−*^ or *Na*_*V*_*1.8Cre-Gpbar1*^*−/−*^ mice (Fig. 8D).

### CCDC-evokes increases in intracellular Ca^2+^ in a small proportion of non-neuronal cells

**Figure 9:**
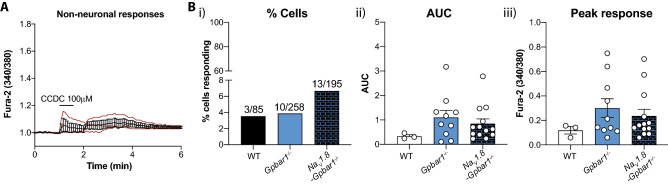
Intracellular calcium responses to CCDC in non-neuronal cells from wildtype, *Gpbar1*^−/−^ and *Na*_*V*_*1.8-Gpbar1*^−/−^ mice. (**A**) Intracellular calcium changes were measured in non-neuronal cells from wildtype (WT: black), *Gpbar1*^*−/−*^ (light blue) and *Na*_*V*_*1.8*-*Gpbar1*^*−/−*^ (dark blue) mice in response to CCDC (100 µM). Mean ± SEM of intracellular Ca^2+^ responses to CCDC (100 μM) in non-neuronal cells from wildtype mice. Red lines represent the three individual responding cells (3/85). (**B**) (**i**) The percentage (%) of non-neuronal cells responding to CCDC (100 µM). 3.5% (3 out of 85), 3.88% (10 out of 258) and 6.67% (13 out of 195) of non-neuronal cells from wildtype, *Gpbar1*^*−/−*^, and Na_V_1.8-*Gpbar1*^*−/−*^ respectively responded to CCDC (100 µM). (**ii**) No significant differences were observed in either the average intracellular calcium response over time or (**ii**) the peak fluorescent response to 100 µM CCDC between non-neuronal cells from wildtype, *Gpbar1*^*−/−*^ (light blue) and Na_V_1.8-*Gpbar1*^*−/−*^ (dark blue) mice. Data are represented as percentage of cells (**Bi**) or Mean ± SEM (**Bii**,**Biii**), with individual data points representing values from individual cells. Significance was determined by Kruskal–Wallis non-parametric analysis of variance with Dunn’s multiple comparisons tests (**Bii**,**Biii**).

We have shown that the effects of CCDC in the intact bladder, namely mechanical hypersensitivity of pelvic afferents and spinal cord activation, is prevented in *Gpbar1*^*−/−*^ mice. Therefore, we investigated if the *in-vitro* effects we observed with CCDC in neurons from *Na*_*V*_*1.8Cre-Gpbar1*^−/−^ and *Gpbar1*^−/−^ mice resulted from additional cell types responding to CCDC that are not present in the bladder (Fig. 9). We observed that a small population (3%) of non-neuronal cells from wildtype mice responded to CCDC (Fig. 9ABi–iii). Surprisingly, non-neuronal responses to CCDC were retained in cells from *Gpbar1*^*−/−*^ and *Na*_*V*_*1.8Cre-Gpbar1*^*−/−*^ mice (Fig. 9Bi). Furthermore, the area under the response curve (AUC, Fig. 9Bii) and the maximum peak of Fura-2 fluorescence in response to CCDC (Fig. 9Biii) were not significantly reduced in cells from *Gpbar1*^*−/−*^ nor *Na*_*V*_*1.8Cre-Gpbar1*^*−/−*^ mice.

## Discussion

The overlapping symptoms of OAB and IC/BPS, including frequency, urgency and discomfort indicate an increase in the sensitivity of bladder afferent neurons to bladder filling. Identifying the receptors and channels involved in bladder afferent neuronal activation, and the impact these have on behavioural responses in states of health and hypersensitivity, is a crucial step to developing efficacious treatment options for patients suffering bladder hypersensitivity disorders. Here, we have identified a role for the bile acid receptor TGR5 in mediating bladder afferent mechanical hypersensitivity in vivo and ex vivo. Intravesical administration of the progesterone metabolite Pg5α and the synthetic TGR5 agonist CCDC increased afferent sensitivity to bladder distension at both peripheral and spinal levels, responses which we show to be reduced or absent in mice lacking the gene for TGR5 (*Gpbar1*).

These findings are important as endogenous TGR5 agonists, including bile acids and progesterone metabolites are present at low levels in the urine of healthy subjects^33,34^ and are increased dramatically under different conditions, including pregnancy and liver disease^32,50^. As such, the increased sensitivity of these mechanosensitive bladder afferents following intravesical administration of Pg5α suggests an endogenous role for TGR5 agonists in mediating afferent hypersensitivity. The CCDC-induced increase in bladder afferent firing to distension largely mirrored that of PG5α, although the sensitisation was observable from earlier distension pressures following CCDC administration compared to PG5α. Importantly, the overall bladder afferent hypersensitivity induced by CCDC was abolished in *Gpbar1*^*−/−*^ mice and the percentage of responding afferents greatly reduced.

These findings of afferent sensitisation by TGR5 agonists have physiological relevance as projections from bladder afferents terminate within the lumbosacral spinal dorsal horn^10,51^. Following peripheral activation, these neurons release neurotransmitters within the dorsal horn which act on second order neurons. Sub-populations of second order spinal neurons involved in bladder sensory pathways have been identified within the superficial dorsal horn (SDH), the dorsal grey commissure (DGC) and the sacral parasympathetic nucleus (SPN)^6,16,36,51–55^. These second order neurons relay peripheral bladder sensory input to the brain, resulting in autonomic micturition reflexes and cognitive perception^6,36,47,56^. Here, we demonstrate in sacral spinal segments that following CCDC and bladder distension there is an increase in the number of pERK-IR neurons within the SDH, the DGC, the lateral spinal nuclei and the SPN. This indicates peripheral bladder afferent hypersensitivity induced by TGR5 agonism is relayed into the spinal cord and subsequent central pathways. These findings correlate well with our previous studies whereby intravesical application of histamine or Mrgpr agonists increases bladder afferent responses to bladder distension, resulting in increased neuronal activation within the spinal cord dorsal horn regions associated with micturition and nociception^16,17^.

Our mRNA expression results confirm *Gpbar1* expression in all layers of the mouse urinary bladder, building on a previous study showing expression of the TGR5 gene *Gpbar1* mRNA in whole rat bladder^23^. Whilst TGR5 is known to play an important role in smooth muscle relaxation, particularly in the gallbladder and the stomach^57–59^, it is particularly important to note that in the current study bladder muscle compliance was unchanged following intravesical administration of either Pg5α or CCDC. This indicates the Pg5α- or CCDC-induced hypersensitivity of bladder afferents to distension occurs at the level of the afferent ending. Importantly our single cell RT-PCR data show *Gpbar1* expression in 13% of bladder-innervating DRG neurons, which is comparable to recent studies in cutaneous- and colon-innervating DRG neurons showing 8% and 19% are positive for *Gpbar1* mRNA expression, respectively^24,26^. Both studies further analysed co-expression with *Trpa1* and *Trpv1*, which are common downstream targets for GPCR signalling and have both been shown to play a role in mediating itch and pain sensation^15,48^. These previous studies indicate that TRPA1 is the key downstream effector coupled to neuronal TGR5, with both cutaneous and colonic afferent hypersensitivity to CCDC abolished in *trpa1*^*−/−*^ mice^24,26^. Previous expression data largely supports this, as cutaneous- and colon-innervating DRG neurons show high co-expression with both *trpa1* (100% in cutaneous and 83% in colonic *Gpbar1*-positive DRG neurons) and *trpv1* (60% in cutaneous and 78% in colonic *Gpbar1*-positive DRG neurons)^24,26^. Another study assessing *Gpbar1* mRNA expression in small diameter DRG neurons showed lower associations with both *trpa1* (41% of *Gpbar1*-positive small diameter DRG neurons) and *trpv1* (32% of *Gpbar1*-positive small diameter DRG neurons)^29^. Interestingly, we show bladder-innervating DRG neurons differ to cutaneous and colon-innervating DRG neurons, with 100% of bladder-innervating *Gpbar1*-expressing DRG neurons co-expressing *trpv1,* but only 30% co-expressing *trpa1*.

Our in vitro functional calcium imaging data showed CCDC activated a population of bladder-innervating DRG neurons, and that the magnitude of these CCDC responses were greater in bladder-innervating neurons than non-bladder innervating neurons, suggesting an enhanced role in bladder pathways. Bladder-innervating DRG neuron responses to CCDC supports previous studies whereby CCDC activates sub-populations of colonic and cutaneous DRG neurons^24,26^. However, unlike these previous studies, significant functional co-expression was observed between TGR5 and TRPV1 (CCDC and capsaicin responsive) with less interaction observed between TGR5 and TRPA1 (CCDC and AITC responsive). These data correspond with our mRNA expression data which demonstrates extensive *Gpbar1 and trpv1 co-expression,* suggesting an alternative downstream coupling mechanism for TGR5 to that seen in colonic or cutaneous DRG neurons. The potential associations of in vitro CCDC activity with TRPA1 and TRPV1 were further dissected using DRG from *trpa1*^*−/−*^ and *trpv1*^*−/−*^ mice. Data from these DRG neurons demonstrate that CCDC-induced activation of bladder-innervating DRG neurons was greatly reduced in *trpv1*^*−/−*^ DRG neurons, whilst no significant differences in CCDC responses were observed in the absence of *trpa1*. Together, these data indicate that TGR5 signalling in bladder afferents is partially mediated by TRPV1 as a potential downstream effector.

Our ex vivo and in vivo data with CCDC are relatively clear cut in the role TGR5 plays in bladder afferent hypersensitivity, with agonist responses reduced or absent in *Gpbar1*^*−/−*^ mice. However, the proportion of bladder-innervating DRG neurons responding to CCDC is greater than the single cell mRNA expression data predicts. Furthermore, in these calcium imaging studies CCDC responses were still observed in DRG from *Gpbar1*^*−/−*^* (global knockout of Gpbar1) and Na*_*V*_*1.8-Gpbar1*^*−/−*^ (neuronal knockout of *Gpbar1* as 98% of bladder-innervating DRG neurons express Na_V_1.8^37^) mice. Whilst CCDC is a synthetic agonist developed to be TGR5-specific^38^ it is unclear if it can also act via additional receptors. For example, in addition to TGR5^28^ progesterone metabolites can act on progesterone receptors and GABA_A_ receptors^60,61^, although GABA_A_ receptor agonists inhibit rather than sensitize visceral afferents^62^. In addition, recent data show that some bile acids can also act through MrgprX4^25,63^. In the current study we also found that CCDC activated a small percentage of non-neuronal cells and that these responses were similar between wildtype, *Gpbar1*^*−/−*^* and Na*_*V*_*1.8-Gpbar1*^*−/−*^ DRG neurons. A recent study has shown in human and monkey that TGR5 is highly expressed in satellite glial cells surrounding DRG neurons but not the DRG neurons, whereas in mouse TGR5 is expressed by DRG neurons^25,63^. It is possible that some of the disparity observed in our DRG calcium imaging studies are related to indirect actions, whereby satellite glial cells release factors in response to CCDC application and that these factors then act on and activate DRG neurons. These discrepancies aside overall our mRNA expression, ex vivo and in vivo functional evidence indicate that the physiological actions of both the synthetic and the endogenous TGR5 agonists are mainly due to activation of TGR5. In particular, the enhanced dorsal horn neuronal activation in response to CCDC and bladder distension is completely lost in *Gpbar1*^*−/−*^ mice, suggesting TGR5 expressing bladder-innervating DRG neurons are crucial for these sensitizing actions of CCDC in vivo.

How bile acid or progesterone metabolite urinary content is altered during bladder hypersensitivity is not yet known. However, an important consideration in determining any role for TGR5 in OAB and IC/BPS is the permeability of the urothelium. Increased urothelial permeability is a common finding in IC/BPS, which may allow increased access of urinary contents, such as bile acids and progesterone metabolites, to access the underlying tissue and nerve endings within the bladder wall^1,64^. Further studies are required to determine the impact of TGR5 signalling in bladder afferents during states of hypersensitivity and any changes specific to this pathway including receptor expression and agonist concentrations in the urine and tissue.

In conclusion, this study has identified the expression and function of the bile acid receptor TGR5 in bladder-innervating afferent DRG neurons. Intravesical administration of TGR5 agonists in the urinary bladder results in TGR5-dependent enhanced mechanosensitivity to bladder distension and increased spinal dorsal horn neuronal activity, indicating a role for TGR5 in mediating the sensitivity of bladder sensory signalling pathways. Further, we have shown that TGR5 activation in bladder-innervating neurons is partially mediated by TRPV1. As irritant-sensing GPCRs are now known to enhance bladder mechanosensitivity, TGR5 and its related receptors may represent potential targets for future treatment in conditions of bladder hypersensitivity.
